# MATNet: multi-level fusion transformer-based model for day-ahead PV generation forecasting

**DOI:** 10.3389/frai.2026.1891798

**Published:** 2026-07-14

**Authors:** Matteo Tortora, Francesco Conte, Gianluca Natrella, Paolo Soda

**Affiliations:** 1 Department of Naval, Electrical, Electronics and Telecommunications Engineering, University of Genoa, Genoa, Italy; 2 Unit of Innovation, Entrepreneurship & Sustainability, Department of Engineering, University Campus Bio-Medico of Rome, Rome, Italy; 3 Artificial Intelligence and Computer Systems, Department of Engineering, University Campus Bio-Medico of Rome, Rome, Italy; 4 Department of Diagnostics and Intervention, Radiation Physics, Biomedical Engineering, Umeå University, Umeå, Sweden

**Keywords:** deep learning, forecasting, multimodal learning, photovoltaic generation, renewable energy source, soft-attention, transformer

## Abstract

Accurate forecasting of renewable generation is crucial to facilitate the integration of Renewable Energy Sources (RESs) into the power system. Focusing on photovoltaic (PV) units, forecasting methods can be divided into two main categories: physics-based and data-based strategies, with Artificial Intelligence (AI)-based models providing state-of-the-art performance. However, while these AI-based models can capture complex patterns and relationships in the data, they rarely exploit the physics-derived information produced by Numerical Weather Prediction (NWP) models. Therefore, in this paper, we propose MATNet, a novel transformer-based multimodal architecture for multi-step day-ahead PV power generation forecasting. The model is fed with historical PV data and historical and future weather covariates through a multi-level joint fusion approach, employing a soft-attention mechanism at multiple fusion stages. We evaluate MATNet on the Ausgrid benchmark dataset. MATNet achieves an RMSE of 0.0445, corresponding to a relative improvement of approximately 65% over the best-performing external baseline. The evaluation includes ablation studies, a sensitivity analysis on missing data, a cross-site zero-shot evaluation on five external PV datasets, and a computational complexity analysis. These experiments assess the contribution of each input modality, the resilience to input degradation, the transferability across PV sites, and the trade-off between accuracy and computational cost. These results highlight MATNet's potential as a reliable and efficient solution to facilitate the integration of PV energy into the power grid. The code is available at https://github.com/arco-group/MATNet.

## 1 Introduction

The integration of renewable energy into power systems is steadily increasing due to the combination of climate change and the consequent need to reduce greenhouse gas emissions. The shift toward RESs is crucial to a sustainable, affordable, accessible, clean, and low-carbon future, reducing polluting emissions and dependence on fossil fuels. In this regard, PV energy is one of the most mature RES technologies, called to play a crucial role in accomplishing various climate protection goals (International Renewable Energy Agency, 2020; International Energy Agency, 2022; Zhou, 2022).

Compared with fossil fuel-derived energy, renewable energy is more sustainable. However, its intermittent nature does not guarantee constant production flows, causing imbalances in the power system that ultimately limit large-scale adoption (Meenal et al., 2022; Simeunović et al., 2021). PV energy generation forecasting could help resolve these imbalances and uncertainties, facilitating the general integration of RES into the power systems (Simeunović et al., 2021; Wan et al., 2015; Gupta and Singh, 2021; Aslam et al., 2021; Kaur et al., 2022b).

Accurately forecasting PV production is essential to realizing the full potential of PV systems and providing grid operators and energy traders with valuable insights and decision-making information to optimize maintenance strategies, plan the development of new plants, mitigate operational and management challenges, and improve economic returns on investment (Alkhayat and Mehmood, 2021; Capotosto et al., 2022; Bianchi et al., 2023; Wan et al., 2015). For this reason, several forecasting methods PV energy production have recently been developed (Iheanetu, 2022). Currently, PV forecasting methods can be divided into physics-based and data-based strategies (Gupta and Singh, 2021). The former, also known as Numerical Weather Prediction (NWP), are mathematical models that simulate complex systems to predict how the atmosphere will evolve. These models are based on fluid dynamics and thermodynamic principles and use a combination of observations from weather stations, satellites, radars, and other sources to predict atmospheric dynamics. However, the main drawback of these methods is their lack of flexibility, as they require intensive knowledge about the considered phenomena and can be expensive in terms of time and resources. Additionally, they require extensive computer resources to run effectively (Wan et al., 2015; Chai et al., 2020). The latter (i.e., data-based approaches) can be further split into statistics- and Artificial Intelligence (AI)-based methods. On the one hand, statistical methods include well-known approaches like linear regression, Auto-Regressive Moving Average or Auto-Regressive Integrated Moving Average, which aim to establish the mathematical framework governing the data-generation process (Barker, 2020). However, despite their prevalent application in the literature and while some incorporate nonlinear approaches, they may not be optimal for uncovering complex nonlinear relationships (Januschowski et al., 2020). On the other hand, AI-based models have dominated research in recent years due to their ability to discover complex nonlinear relations, deal with unstructured data, and superior performance. Compared with the former methods, they allow for easier modeling without requiring prior knowledge of the phenomenon's dynamics (Li et al., 2023). In particular, Deep Learning (DL) has shown promising results in forecasting PV energy production due to its ability to generalize and automatically extract abstract representations, and recurrent neural networks (RNNs) as well as convolutional neural networks (CNNs) are the most used DL-based architectures in PV power forecasting (Aslam et al., 2021). However, despite their advantages, DL methods often rely on a single data source and do not fully exploit the domain-specific physical information that could enhance their accuracy. For instance, in many PV forecasting models, weather conditions, which directly influence energy production, are not provided as explicit input. NWP-based methods, in contrast, excel at incorporating this knowledge, albeit with limitations in scalability and flexibility. This dichotomy highlights the need for frameworks that can integrate diverse data modalities, leveraging NWP outputs as physics-derived inputs within a data-driven architecture.

Multimodal learning offers a promising solution to address these challenges by integrating heterogeneous data sources to enhance predictive performance beyond the limitations of stand-alone modalities, demonstrating notable success across diverse fields, ranging from medical diagnostics to natural language processing (Tortora et al., 2023; Furia et al., 2023; Mulero Ayllón et al., 2026; Coser et al., 2025; Cordelli et al., 2020), despite the absence of formal theoretical proof. Multimodal learning can be implemented at different levels through three fusion strategies: early fusion, joint fusion, and late fusion (Baltrušaitis et al., 2018; Ramachandram and Taylor, 2017). Early fusion involves combining raw data from multiple modalities at the input stage, allowing the model to learn joint feature representations from the beginning. While straightforward, this approach faces challenges when handling inputs with varying scales or temporal resolutions, requiring the model to account for these differences during training. Joint fusion, on the other hand, integrates information at an intermediate level, combining features extracted separately from each modality. This approach preserves the unique characteristics of each modality while learning shared representations, making it particularly effective for capturing complex, nonlinear interdependencies between data sources. In contrast, late fusion aggregates outputs from models trained independently on each modality at the decision-making stage. While this strategy is simpler and can reduce overfitting risks, it may miss opportunities to capture deeper interdependencies between modalities during training. For an in-depth discussion of these fusion techniques, see Baltrušaitis et al. (2018).

The adoption of multimodal learning can facilitate the development of robust and accurate PV forecasting models by integrating the complementary strengths of physics- and data-driven approaches. Nevertheless, its application in PV forecasting remains relatively underexplored (Li et al., 2023; Bai et al., 2022). To fill this gap, in this work, we propose MATNet, a novel multimodal self-attention-based architecture for multi-step, day-ahead PV power production forecasting, combining the advantages of a DL approach with the physics-derived meteorological covariates produced by NWP models. The contributions of this work are summarized as follows:

- We propose MATNet, a novel transformer-based multimodal architecture for multi-step day-ahead PV generation forecasting, incorporating three data modalities: PV production data, weather history conditions, and future weather covariates.
- We introduce a multi-level joint fusion framework integrating heterogeneous data sources through a multi-stage soft-attention mechanism. By leveraging the temporal distinction between historical and future meteorological covariates, the framework dynamically adjusts contributions at different stages, effectively capturing cross-modal dependencies and enhancing predictive accuracy.
- Inspired by interpolation techniques from language modeling, we propose a dense interpolation layer that generates concise representations while preserving temporal structure from high-dimensional attention-based outputs. This layer adaptively learns weights for interpolating hidden states, capturing the temporal dynamics of time-series data and enhancing the flexibility of the final representation.
- We evaluate the model's effectiveness on the Ausgrid benchmark dataset (Ausgrid, 2014), comparing its performance against thirteen baseline models, including standard statistical approaches, machine learning methods, and deep neural networks. The analysis is further extended through ablation studies, a sensitivity analysis on missing data, a cross-site zero-shot generalization evaluation on five external PV datasets, and a comprehensive assessment of the model's computational complexity.

The rest of this manuscript is organized as follows: section 2 presents a review of related work. section 3 introduces the datasets and the adopted processing steps. section 4 describes the details of our approach, whilst section 5 describes the experimental setup and discusses the results. Finally, section 6 provides concluding remarks.

## 2 Related work

This section focuses on works based on the Ausgrid dataset (Ausgrid, 2014), which contains historical energy production data from 300 residential PV units. subsection 3.1 describes this dataset in detail.

In Fentis et al. (2020) the authors proposed an ensemble approach for 24-hour-ahead PV generation forecasting. The idea behind this is to break down the PV production time series into sub-time sequences, so that each of these sub-time series collects PV power records for the same time and all days of the year, creating a time series for every 30 minutes of production. Therefore, their approach consists of an ensemble of simple univariate models based on Least Squares Support Vector Regression (LsSVR), each of which predicts the PV output of each 30 minutes of the following day. They demonstrated that the proposed approach is generic and any machine learning algorithm for regression can be used. Although the model performs well on *clear* and *partly cloudy* days, it struggles under *cloudy* and *overcast* conditions.

Building on the limitations of traditional machine learning models, in Kaur et al. (2021) the authors introduce a Bayesian probabilistic method incorporating Bidirectional Long Short-Term Memory (BiLSTM) networks. This model addresses uncertainties in PV generation data and model parameters, achieving multi-step ahead forecasts with improved probabilistic accuracy. Results demonstrated its effectiveness over other methods by evaluating probabilistic metrics like Pinball loss and Winkler score, underscoring the model's robustness. Building on this, in a subsequent work (Kaur et al., 2022a) the authors proposed an improved Bayesian bi-directional short-term memory (BiLSTM)-based DL technique integrating an alpha-beta divergence, specifically designed to handle outliers in PV generation data. The results demonstrate that this improved Bayesian BiLSTM with alpha-beta divergence outperforms standard Bayesian BiLSTM and other benchmark methods for multi-step ahead forecasting in terms of lower error values, leading to better error reduction and robustness against data anomalies. Lastly, the same authors proposed a further enhancement in (Kaur et al., 2023), which combines Bayesian BiLSTM with variational autoencoders to tackle the high dimensionality in weight parameters. This approach for PV forecasting reduces computational overhead by compressing the dimensionality of the parameter space. The model's efficiency was demonstrated with significant reductions in computational time and improved forecasting accuracy, thus providing a scalable solution for renewable energy forecasting with uncertain, high-dimensional data.

These approaches, while effective in certain conditions, reveal notable limitations under adverse weather conditions tied to their underlying unimodal methodologies. Unimodal models restrict their focus to a single data source, such as historical PV power production, without incorporating complementary data sources. To fill these gaps, we propose a novel multimodal architecture for multi-step, day-ahead PV forecasting, integrating heterogeneous sources such as historical PV production data, weather history, and weather forecasts within a unified framework that leverages advanced attention mechanisms to capture intricate temporal and cross-modal dependencies among these data streams. This multimodal approach not only enhances the model's ability to adapt to diverse weather conditions but also improves accuracy and robustness in forecasting under varying weather scenarios.

## 3 Materials

In this work, we utilize three primary data sources for multi-step PV power production forecasting: Ausgrid, OpenWeatherMap, and Solcast. Ausgrid is the electricity distributor for New South Wales, Australia, and provides a dataset on historical electricity demand and PV power generation. OpenWeatherMap is an online weather data service that provides real-time, forecast, and historical weather data. Lastly, Solcast is an online solar data service providing forecasts, live and historical solar irradiance, as well as weather data, which is freely available for public research purposes or implementation in household systems. In the following, we will describe in further detail the characteristics and features of these datasets, as well as the pre-processing steps used in this study.

### 3.1 Ausgrid

**Figure 1:**
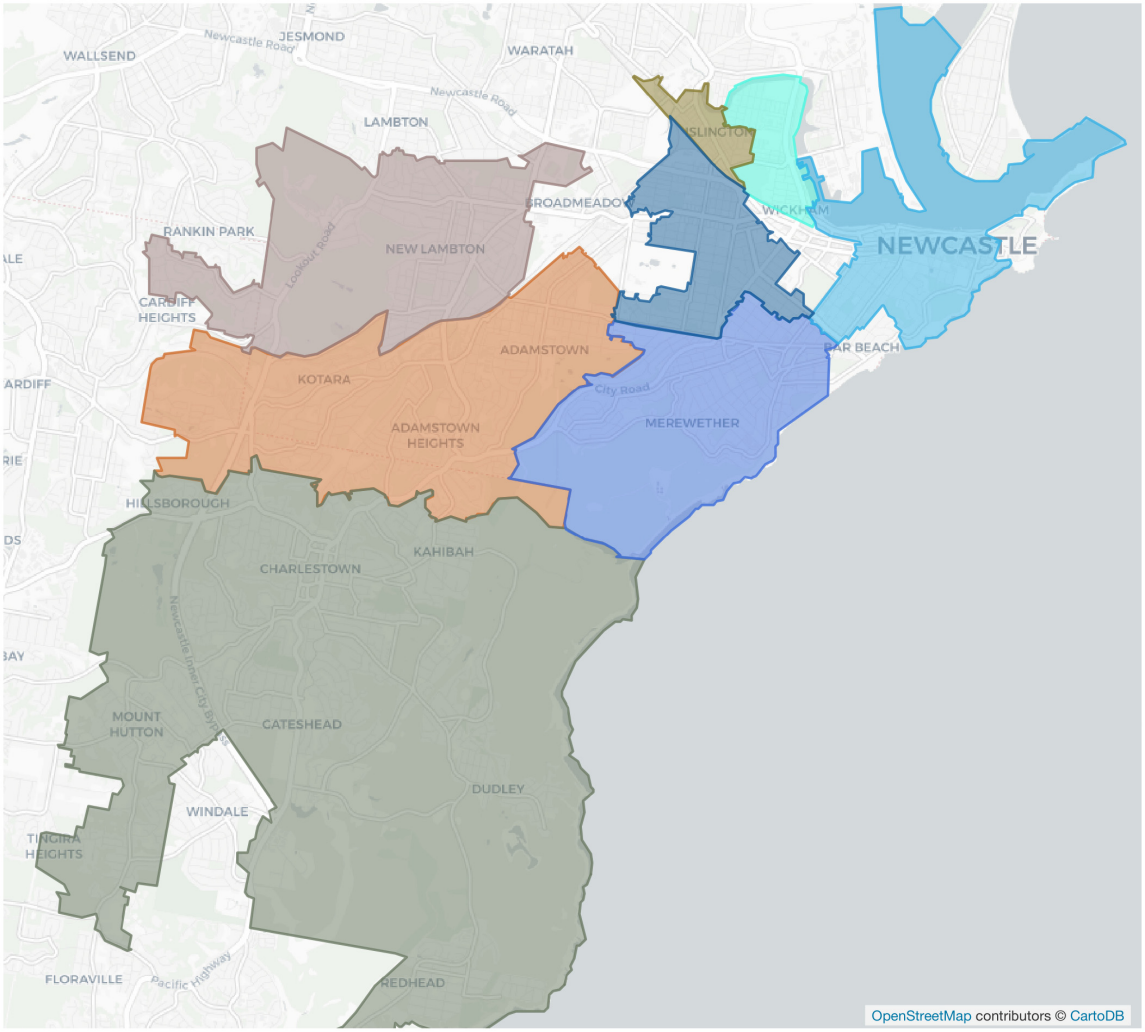
Map of the territorial areas containing the 26 PV units considered in this study, generated using the PyTrack library (Tortora et al., 2022). Each colored area corresponds to a distinct geographical region associated with one of the eight zip codes. The study area falls within the Cfa (humid subtropical) climate zone according to the Köppen-Geiger classification. Map data available from OpenStreetMap, https://www.openstreetmap.org/copyright, under the Open Data Commons Open Database License [ODbL].

We utilized the “Solar home electricity dataset” provided by Ausgrid (Ausgrid, 2014) as the primary source of data. This electricity distributor provider owns, maintains, and operates the electricity distribution network in Sydney, the Central Coast, and the Hunter regions of New South Wales, Australia. The dataset contains energy generation (in kWh) for 300 residential rooftop solar PV units recorded directly from the PV inverter over the period from 1 July 2010 to 30 June 2013 with a sampling time of 30 minutes. To ensure data integrity, we selected only 26 out of 300 households with PV units spread over a 75-square-kilometer area and located in 8 different zip codes within the Newcastle region (as illustrated in Figure 1), which falls within the Cfa (humid subtropical) climate zone according to the Köppen-Geiger classification; a comprehensive list of the included customers is reported in Appendix A. While this filtering ensures high-quality and consistent measurements, it may also introduce a mild selection bias toward PV systems with more stable operation, slightly limiting the dataset's representativeness under more heterogeneous conditions.

We pre-processed the data as follows. Let *X* = {*x*_*i*_(*t*_*m*_) ∣ *i* = 1, …, *N*; *m* = 1, …, *T*} denote the collection of PV energy time series for *N* households over *T* time steps, with a sampling interval Δ*t* = 30 minutes. Here *x*_*i*_(*t*_*m*_) represents the energy generated over the half-hour interval (*t*_*m*−1_, *t*_*m*_]. First, we aggregated the series to an hourly sampling frequency by summing the two half-hour values that fall within each hour, a choice motivated by the need to align with practical energy forecasting applications such as day-ahead scheduling and market bidding (Perera et al., 2022; ENTSO-E, 2022; Exchange, 2020), and supported by a granularity analysis (Appendix B) showing stable performance from 30 minutes to 6 hours, with the 1-hour resolution as the best trade-off. Let τ_*k*_ = *t*_2*k*−2_ denote the start time of the *k*-th hour and let $\tilde{T}=\left\lfloor T/2\right\rfloor$ be the number of hourly steps. The aggregated hourly series for the *i*-th household is:


$$\begin{array}{l} {\tilde{x}}_{i}\left(\tau_{k}\right)=\overset{2}{\underset{j=1}{\sum }}=x_{i}\left(t_{2k-2+j}\right),\;k=1,2,\ldots ,\tilde{T}. \end{array}$$ (1)

Each value ${\tilde{x}}_{i}\left(\tau_{k}\right)$ therefore corresponds to the energy generated in the hour (τ_*k*_, τ_*k*_+1*h*]. Next, we aggregated the subsampled PV facilities' data across all households by summing the energy production of each household at every timestamp:


$$\begin{array}{l} {\tilde{P}}_{agg}\left(\tau_{k}\right)=\overset{N}{\underset{i=1}{\sum }}{\tilde{x}}_{i}\left(\tau_{k}\right),\;k=1,2,\ldots ,\tilde{T} \end{array}$$

To improve numerical stability during model training, we normalized the aggregated time series by the total installed capacity of all households, represented as the sum of the kilowatt-peak (*kWp*) of each household, yielding the hourly specific yield:


$$\begin{array}{l} \bar{P}\left(\tau_{k}\right)=\frac{{\tilde{P}}_{agg}\left(\tau_{k}\right)}{\sum_{i=1}^{N}kWp_{i}},\;k=1,2,\ldots ,\tilde{T} \end{array}$$

**Figure 2:**
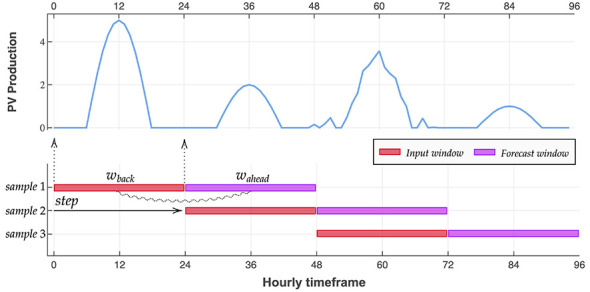
Sliding window scheme for day-ahead PV forecasting. Red and purple bars denote the input (*w*_*back*_) and forecast (*w*_*ahead*_) windows, respectively, shifted over time with a fixed *step*.

This normalization not only ensures that all values are scaled between 0 and 1, but also leads to better conditioning of the problem and faster learning during the training phase, facilitating a more efficient optimization process (LeCun et al., 2002). Finally, to prepare the data for model training, we extracted samples using a sliding window parameterized by two factors: the width of the sliding block (*w*) and the step size (*step*) between windows. Each window serves as an input sample, paired with target values corresponding to the aggregated PV energy production for the subsequent 24-hour period. A schematic illustration of the sliding window extraction process is provided in Figure 2. The specific values of the parameters are detailed in Section 5.1. For the sake of simplicity in the subsequent text, we denote the pre-processed PV power production time series as $\mathrm{pv}\in {\mathbb{R}}^{w_{\text{back}}\times 1}$, representing the historical PV power production over a look-back window of *w*_back_ time steps.

### 3.2 Weather conditions

We utilized as the primary source for meteorological data OpenWeatherMap service (OpenWeather Ltd., 2023), which provides weather data for any location using an NWP proprietary model. It is fed with 82,000 weather stations spread globally, radars, and weather satellites.

In addition to the meteorological data obtained from OpenWeatherMap, we incorporated solar radiation data from Solcast (Solcast, 2019). We incorporated the following irradiance measurements to characterize the availability and intensity of solar energy at the considered location:

- Direct Normal Irradiance (DNI): It represents the direct irradiance received on a surface held perpendicular to the sun, providing information about solar radiation intensity when the sunlight reaches the surface directly without any obstructions.
- Diffuse Horizontal Irradiance (DHI): It refers to the diffuse irradiance received on a horizontal surface. It quantifies the scattered radiation caused by atmospheric factors such as clouds, haze, and pollution.
- Global Horizontal Irradiance (GHI): This measurement represents the irradiance received on a horizontal surface. It takes into account both direct sunlight and diffuse sky radiation and is crucial for assessing the solar energy potential of a location.

Table 1 summarizes the climate attributes provided by both the OpenWeatherMap API and Solcast considered in this work. Unlike all other variables which are numerical features, the *weather description* attribute is a categorical feature with 22 distinct levels, listed in Appendix C. In order to convert these categorical values into numeric ones we apply a one-hot encoding, as no ordinal relation exists for these variables.

**Table 1:** Weather attributes extracted from OpenWeather API and Solcast.

Attribute	UoM	Description
Temperature	K	Temperature
Pressure	hPa	Atmospheric pressure (on the sea level)
Humidity	%	Humidity
Dew point	K	Temperature at which condensation occurs
Wind speed	ms^−1^	Wind speed
Wind deg	deg	Wind direction
Clouds all	%	Cloudiness
Rain 1h	mm	Rain volume for the last hour
Weather description	-	Categorical weather conditions
Direct Normal Irradiance (DNI)	Wm^−2^	Direct Normal Irradiance
Diffuse Horizontal Irradiance (DHI)	Wm^−2^	Diffuse Horizontal Irradiance
Global Horizontal Irradiance (GHI)	Wm^−2^	Global Horizontal Irradiance

As for the power production data, we generated meteorological samples using the same sliding-window scheme. The input weather history was defined from the observations available in the look-back window. The future weather branch requires meteorological covariates over the following 24 hours. However, historical operational forecasts for the 2010–2013 period were not available from OpenWeatherMap or Solcast. For this reason, we did not use true archived weather forecasts. Instead, we constructed forecast-like weather inputs from the corresponding future meteorological records. This choice should be interpreted as a controlled experimental proxy rather than as a replacement for archived operational forecasts. It allows the model architecture to be evaluated under the intended multimodal setting, where past PV production, past weather, and future meteorological covariates are provided as separate input streams. At the same time, it removes the forecast-model error that would be present in a real deployment. To partially account for this gap, we perturbed the future meteorological covariates with additive Gaussian noise. In the main experiments, we used a 5% noise level. This value was not intended to model a specific forecasting provider, but to avoid evaluating the future-weather branch under error-free conditions. To assess the dependence of MATNet on this assumption, we also performed a sensitivity analysis by varying the noise level from 0% to 20% in increments of 5%. The results, reported in Appendix D, show a gradual degradation as the perturbation magnitude increases, with limited changes in RMSE and MASE across the tested range. This analysis does not replace an evaluation with archived operational forecasts. It provides a robustness check showing that the reported performance is not tied to a noise-free future-weather input. At the end of this pre-processing step, we obtained two meteorological time series with 33 attributes: $hw\in {\mathbb{R}}^{w_{\text{back}}\times 33}$, representing the historical weather data over the look-back window, and $fw\in {\mathbb{R}}^{w_{\text{ahead}}\times 33}$, representing the future weather covariates over the look-ahead window.

## 4 Methods

This section presents MATNet, our multi-level fusion and self-attention transformer-based model for multimodal multi-step day-ahead pv forecasting, fed with historical pv production, historical weather data, and future weather covariates.

**Figure 3:**
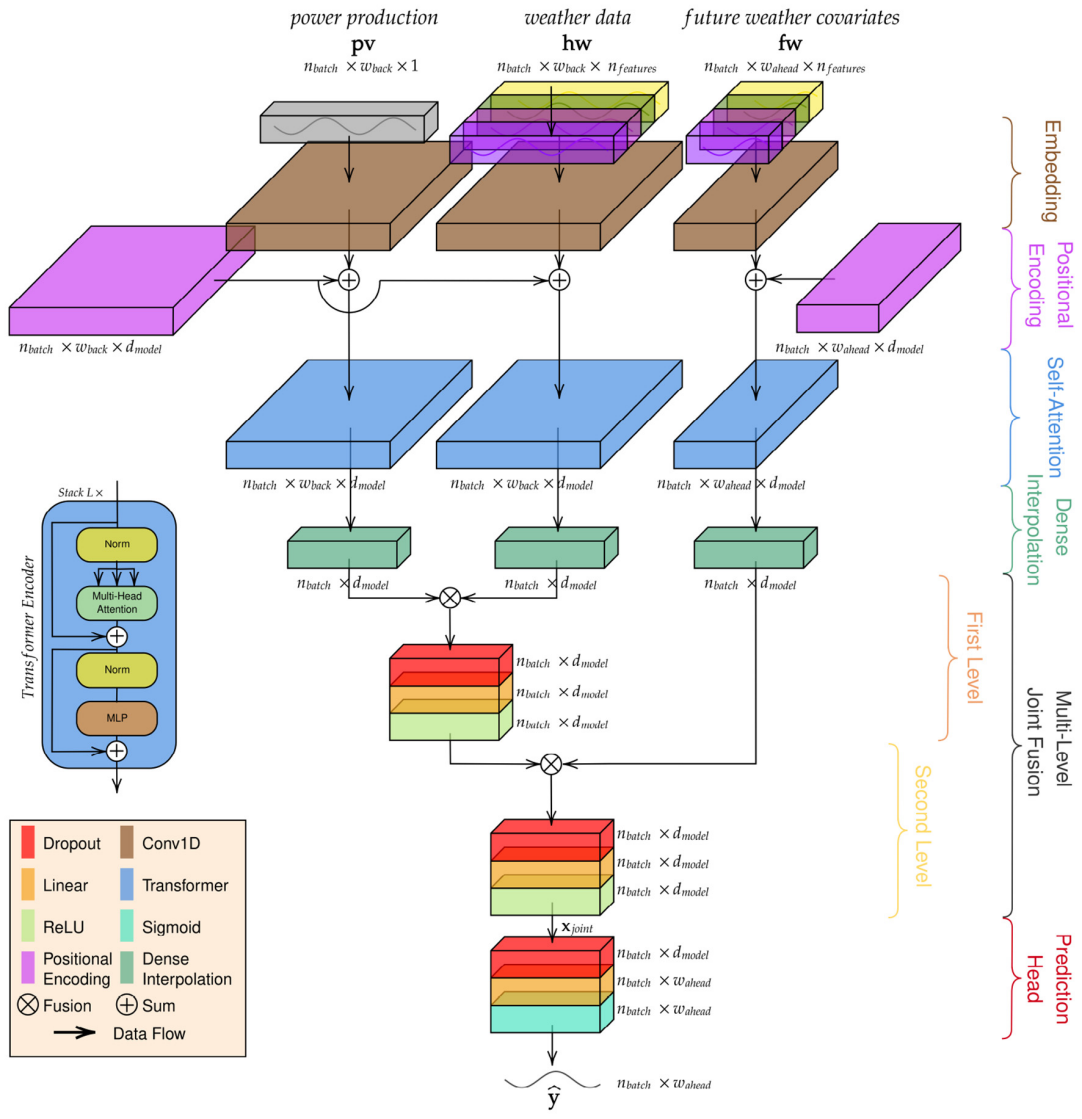
Schematic architecture of our proposed MATNet.

The proposed architecture, depicted in Figure 3, is composed of several key components: embedding modules that project input time series into high-dimensional representations, positional encoding layers to encode temporal order, self-attention modules to highlight relevant features within the sequence, dense interpolation layers that consolidate temporal structures, and a multi-level joint fusion framework that dynamically combines representations from different modalities using a soft-attention mechanism, culminating in the final multimodal vector for PV power prediction. The following sections provide an in-depth description of these components, progressing systematically from the top to the bottom of the architecture, explaining their roles and interactions in achieving the model's objectives.

### 4.1 Embedding module

Our architecture accepts as input a 3-tuple of multimodal time series of the type 〈***pv***, ***hw***, ***fw***〉, where $\mathrm{pv}\in {\mathbb{R}}^{w_{back}\times 1}$, $\mathrm{hw}\in {\mathbb{R}}^{w_{back}\times 33}$ and $\mathrm{fw}\in {\mathbb{R}}^{w_{ahead}\times 33}$ represent the historical PV power production, the historical weather data and the future weather covariates of the next 24-hour time steps to be predicted, respectively, with *w*_*back*_ and *w*_*ahead*_ denoting the look-back and look-ahead windows. The specific values of *w*_*back*_ and *w*_*ahead*_ are detailed in subsection 5.1. The first step in our architecture is an embedding module that maps the multivariate time series into a high-dimensional representation space to facilitate the sequence modeling. This projection enables the model to capture complex relationships within the data that may not be evident in the original input. To this end, we implement a 1D convolutional layer as an embedding module to project the input data to a *d*_*model*_-dimensional space:


$$\begin{array}{l} o_{i}=b_{i}+\overset{C_{in}-1}{\underset{k=0}{\sum }}w_{i,k}*{\mathrm{in}}_{i,k}\;\forall i\in \left\{0,\ldots ,C_{out}-1\right\} \end{array}$$

where ***w*** ∈ ℝ^1 × *size*^ is the convolution kernel, *C*_*in*_ and *C*_*out*_ are the input and the output channels, ***b*** is the bias term, * is the sliding dot product operator (brown block in Figure 3).

When working with PV production data (*C*_*in*_ = 1), we use a convolutional layer with a kernel size of 3 to capture short-term temporal dependencies while preserving the local structure of the data. However, when analyzing weather data (*C*_*in*_ = 33), we use a kernel size of 1, allowing the convolutional layer to focus on capturing dependencies across different time series attributes without considering temporal information. In both cases, the number of output channels is set to *C*_*out*_ = *d*_*model*_. The specific value of *d*_*model*_ is detailed in subsection 5.1.

In conclusion, the output of the embedding module produces multimodal time series with the same number of time steps as the input, but in a higher-dimensional representation with *d*_*model*_ attributes. These representations are then used as input for the attention mechanism described in subsection 4.3.

### 4.2 Positional encoding

As our architecture does not contain any recurrence, we include a positional encoding layer to incorporate the relative or absolute position of the time steps within the input-embedded sequence. This is particularly important for sequential data, as the ordering of the elements can carry valuable information allowing the model to better capture the temporal dependencies in the data and improve the performance of the network.

There are various choices for positional encodings, such as randomized lookup tables or learnable representations (Gehring et al., 2017). Here we use sine and cosine functions of different frequencies as in Vaswani et al. (2017), because they provide a smooth, continuous representation of positions and naturally encode relative distances between time steps, which is beneficial for capturing temporal relationships:


$$\begin{array}{l} {\vec{p_{t}}}^{\left(i\right)}:=\{\begin{array}{ll} \sin\omega_{k}\cdot t, & \text{if}i=2k\;\\ \cos\omega_{k}\cdot t, & \text{if}i=2k+1\; \end{array}, \end{array}$$

where:


$$\begin{array}{l} \omega_{k}=\frac{1}{10000^{\frac{2k}{d_{model}}}} \end{array}$$

and ${\vec{p_{t}}}^{\left(i\right)}$ represents the positional encoding for the *t*-th time step in the input sequence along the *i*-th dimension, and *d*_*model*_ is the input embedding dimension. The *d*_*model*_-dimensional positional embedding is then added to the input embedding since they share the same dimension (light purple block in Figure 3).

### 4.3 Self-attention

Our MATNet architecture relies on self-attention mechanisms to process sequential input data. Self-attention, also known as intra-attention, allows the model to attend to different parts of the whole input sequence, weighing the importance of each component at each position. Unlike recurrent layers that process input data sequentially using recurrent connections and maintaining hidden states to propagate information through time, the self-attention mechanism processes the entire input sequence in parallel, weighing the importance of each element independently (Vaswani et al., 2017; Hochreiter and Schmidhuber, 1997).

An attention function maps a query and a set of key-value pairs to an output, and it is mathematically defined as (Vaswani et al., 2017):


$$\begin{array}{l} \text{Attention}\left(Q,K,V\right)=\text{softmax}\left(\frac{QK^{T}}{\sqrt{d_{model}}}\right)V \end{array}$$

where *d*_*model*_ is the dimension of the key vectors, and **Q**, **K** and **V** are all linear transformations of the input embedding with the added positional encodings. We extended the attention mechanism using multi-head attention: instead of performing a single attention function with keys, values, and queries *d*_*model*_-dimensional matrices, the attention mechanism is split into *h* distinct heads, each operating on an independent projection of the input data. Specifically, the input embeddings are linearly projected into *h* lower-dimensional subspaces. These *h*-projected representations feed the attention mechanisms in parallel, yielding *d*_*v*_-dimensional output values allowing the model to attend to information from different representation sub-spaces at different positions (with *d*_*v*_ = *d*_*model*_/*h*). Each head can be interpreted to encode complementary details on different types of temporal dependencies (Song et al., 2018). Finally, heads are concatenated and linearly projected to obtain the final representation:


$$\begin{array}{l} \begin{array}{ll} \text{MultiHead}\left(Q,K,V\right) & =\text{Concat}\left(head_{1},\ldots ,head_{h}\right)W^{O}\\ \text{where }head_{i} & =\text{Attention}\left(QW_{i}^{Q},KW_{i}^{K},VW_{i}^{V}\right) \end{array} \end{array}$$

where $W_{i}^{Q}\in {\mathbb{R}}^{d_{model}\times d_{k}}$, $W_{i}^{K}\in {\mathbb{R}}^{d_{model}\times d_{k}}$, $W_{i}^{V}\in {\mathbb{R}}^{d_{model}\times d_{v}}$, and $W^{O}\in {\mathbb{R}}^{hd_{v}\times d_{model}}$ are the learnable projections matrices for *i*-th head and for the output, respectively. In this work, we use the same dimension for the queries, keys and values *d*_*k*_ = *d*_*v*_ = *d*_*model*_/*h*. The specific value of *h* is detailed in subsection 5.1.

The second block of the attention module is a simple feed-forward network downstream of the multi-head attention layer. A normalization layer is applied before each block, and residual connections are applied after each block (Dosovitskiy et al., 2020; Baevski and Auli, 2018).

This attention module is stacked *L* times, and the final representations of the entire sequence are obtained from the last one (light blue block in Figure 3).

### 4.4 Dense interpolation layer

Unlike recurrent layers that produce a hidden state summarizing the information from the entire multivariate input sequence (Hochreiter and Schmidhuber, 1997), the attention-based module returns a high-dimensional representation. To create a concise representation while capturing temporal structure and preserving temporal order, we introduce a dense interpolation layer (DIL) (aquamarine block in Figure 3). This layer is inspired by an interpolation algorithm originally developed for language modeling (Trask et al., 2015) and later applied to time-series data (Song et al., 2018). The idea is to interpolate between the attention module's hidden representations ${\text{s}}_{t}\in {\mathbb{R}}^{d_{model}}$ at each time step $t\in \left\{1,\ldots ,\tilde{T}\right\}$ with $\tilde{T}$ being the total number of time steps in the input sequence, weighted by a function of their relative position in the final representation of length *M*:


$$\begin{array}{l} w_{t,m}={\left(1-\frac{\left|s_{t}-m\right|}{M}\right)}^{2},\forall t\in \left\{1,\ldots ,\tilde{T}\right\}\text{ and }m\in \left\{1,\ldots ,M\right\} \end{array}$$

where $s_{t}=M\cdot \frac{t}{\tilde{T}}$ represents the relative position of time step *t* in the final representation, and *m* is the index in the final representation. This weight reflects the contribution of *s*_*t*_ to the final representation at position *m*, decaying quadratically as the relative distance increases, ensuring the temporal structure is captured in the final output. This interpolation is efficiently implemented by performing the following matrix operation: **U** = **S**×**W**, where $\text{S}=\left[{\text{s}}_{1},\ldots ,{\text{s}}_{\tilde{T}}\right]\in {\mathbb{R}}^{d_{model}\times \tilde{T}}$ is the matrix containing the hidden states, $\text{W}\in {\mathbb{R}}^{\tilde{T}\times M}$ stores the interpolation weights *w*_*t, m*_, and $\text{U}\in {\mathbb{R}}^{d_{model}\times M}$ is the final interpolated output representation.

**Figure 4:**
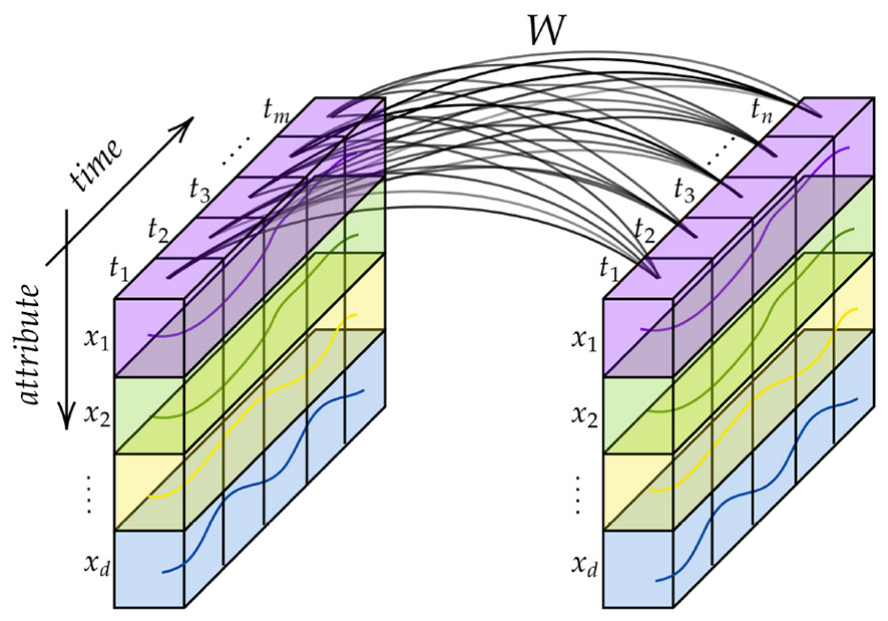
Dense interpolation layer. Here, *m*, *n*, and *d* denote the length of the time series, the interpolation factor, and the number of time-series attributes, respectively. In this work, we have that *m* = *step*_*in*_, *d* = *d*_*model*_ and *n* = *M*.

We further enhance the DIL by allowing the weights *w*_*t, m*_ to be learned via the back-propagation process, enabling a more flexible and adaptive representation. Figure 4 depicts this dense interpolation layer, where edge colors correspond to the magnitude of the learned weights, with darker edges representing stronger contributions and lighter edges indicating weaker ones. Finally, the unified hidden representation can be obtained by several methods, such as concatenation across all time steps. In this work, we use the last embedding as the final representation, summarizing the entire input sequence via the DIL.

### 4.5 Multi-level joint fusion

The dense interpolation stage leads to the generation of three representation vectors in ${\mathbb{R}}^{d_{model}}$, each representing the PV production history, weather history, and future weather branches, respectively. To effectively combine these branch-specific unimodal representations into a unified multimodal vector, we use a fusion layer, also named shared representation layer. Traditional fusion approaches, such as element-wise summation, mean, or Kronecker product, operate at the element-wise or tensor level, applying uniform operations to combine information across modalities. While these methods are effective for some tasks, they often do not fully account for the varying relevance of each input branch and fail to fully capture the intricate interactions and dependencies that exist between heterogeneous data sources, especially when the data streams represent temporally or spatially distinct phenomena (Baltrušaitis et al., 2018), as in our case. To address these limitations, we propose a soft-attention mechanism to guide the fusion process. This attention mechanism helps emphasize the important features within each representation, contributing more effectively to the final multimodal vector. Soft-attention mechanisms have been widely adopted in various tasks, where selective focus has been shown to significantly improve model performance by enabling the model to prioritize informative features while ignoring less relevant ones (Bahdanau, 2014; Vaswani et al., 2017).

Formally, let ${\text{x}}_{1},{\text{x}}_{2}\in {\mathbb{R}}^{d_{\text{model}}}$ represent two input vectors from different modalities. Each input vector is passed through a fully connected layer followed by a non-linear activation function to obtain an intermediate representation:


$$\begin{array}{l} {\text{h}}_{i}=\tanh\left(\text{W}{\text{x}}_{i}+\text{b}\right),\;i=1,2 \end{array}$$

where $\text{W}\in {\mathbb{R}}^{d_{attn}\times d_{model}}$ and $\text{b}\in {\mathbb{R}}^{d_{attn}}$ are learnable parameters. To assess the relevance of each branch, an importance score *s*_*i*_ is computed as the inner product between the intermediate representation ***h***_*i*_ and a learnable context vector $u\in {\mathbb{R}}^{d_{\text{attn}}}$, capturing modality-specific weighting information:


$$\begin{array}{l} s_{i}={\text{u}}^{T}{\text{h}}_{i},\;i=1,2 \end{array}$$

The normalized importance weights α_*i*_ for each branch are then obtained using the softmax function, ensuring that the weights are non-negative and sum to 1, thus providing an interpretable probability distribution over the branches (Bishop and Nasrabadi, 2006; Goodfellow et al., 2016):


$$\begin{array}{l} \alpha_{i}=\frac{\text{exp}\left(s_{i}\right)}{\sum_{k=1}^{2}\text{exp}\left(s_{k}\right)},\;i=1,2 \end{array}$$

where α_*i*_ denotes the contribution of the *i*-th branch to the final representation. Finally, the output of the soft-attention mechanism, denoted by **x**_*attn*_, is computed as the weighted sum of the input vectors:


$$\begin{array}{l} {\text{x}}_{attn}=\overset{2}{\underset{i=1}{\sum }}\alpha_{i}{\text{x}}_{i} \end{array}$$

This formulation allows the model to dynamically adjust the contributions of each input modality, ensuring that the resulting multimodal representation captures both temporal and cross-modal dependencies effectively. Such an approach is particularly advantageous in our scenario, where the input branches represent different temporal aspects of the same phenomenon, including historical PV production and weather data, as well as future weather inputs.

Given that the input modalities observe the phenomenon at distinct points in time, with historical data describing past conditions and future weather covariates describing the look-ahead window, we implement a multi-level fusion approach to integrate information at varying levels of abstraction (black block in Figure 3). At the first fusion level, the two temporally correlated sequences, PV production history and weather history, are fused using the soft-attention mechanism previously described (orange block in Figure 3). The resulting fused vector is then processed through a fully connected module consisting of a dropout regularization layer (Srivastava et al., 2014) and a dense layer with ReLU as an activation function. At the second fusion level, this hidden intermediate representation is then fused with the future weather representation from the dense interpolation layer using another soft-attention mechanism (yellow block in Figure 3). This joint representation is subsequently processed by a fully connected module comprising a dropout layer and a dense layer with ReLU activation, resulting in the unified multimodal representation denoted as:


$$\begin{array}{l} {\text{x}}_{joint}\in {\mathbb{R}}^{d_{model}} \end{array}$$ (2)

which serves as the input to the prediction head described in subsection 4.6. This hierarchical scheme allows the model to progressively integrate historical and forecast information while retaining the most informative features from each modality.

### 4.6 Prediction head and physical constraints

The prediction head (red block in Figure 3) receives the multimodal representation ***x***_joint_ and maps it to the normalized PV production sequence. This module consists of a dropout layer, a linear projection to match the forecast horizon *w*_*ahead*_, and a sigmoid activation function. Formally, the normalized prediction vector is expressed as:


$$\begin{array}{l} \hat{y}=\sigma \left(Linear\left(Dropout\left(x_{joint}\right)\right)\right),\;\hat{y}\in {\left[0,1\right]}^{w_{ahead}} \end{array}$$

where σ(·) is the sigmoid function.

This design inherently enforces two physical constraints: predictions are non-negative and upper-bounded by 1, consistent with the normalized PV time series introduced in subsection 3.1. Together with the NWP-derived input covariates described in subsection 3.2, these constraints represent the two channels through which physical knowledge enters MATNet. In this way, the predicted values directly correspond to normalized specific yields. If needed, forecasts in physical units can be obtained by rescaling with the aggregated installed capacity:


$$\begin{array}{l} \hat{P}=\hat{y}\cdot \overset{N}{\underset{i=1}{\sum }}kWp_{i} \end{array}$$

## 5 Experimental setup and results

In this section, we initially present the experimental setup describing the training details and the evaluation metrics adopted. Then, we introduce thirteen competitors, including statistical methods, methods specific to the Ausgrid dataset (Fentis et al., 2020; Kaur et al., 2021, 2023, 2022a), which are already presented in section 2, and deep neural networks. Finally, we present and discuss the results.

### 5.1 Training details

We use as initial dataset PV power production and weather records from 1 July 2010 to 30 June 2012, splitting it into an 80-20 ratio to create the training and validation sets. The test set contains timestamps ranging from 1 July 2012 to 30 June 2013.

To ensure robustness, reproducibility, and fair comparison, no hyperparameter tuning was performed for MATNet, as hyperparameter optimization is highly dataset-specific and was considered out of the scope of this work. This choice is also consistent with the *No Free Lunch* theorems for optimization, which state that no single algorithm or parameter configuration can be optimal across all possible problem domains (Wolpert and Macready, 2002). In line with this theoretical result, empirical evidence has further shown that hyperparameter tuning does not necessarily yield substantial improvements over default configurations (Arcuri and Fraser, 2013). Unless otherwise specified, we use the following parameters for our architecture: input embedding dimensionality *d*_*model*_ = 512, parallel heads attention layers *h* = 8, number of sub-encoder-layers *L* = 3, the interpolation factor in the interpolation layer *M* = *w*_*back*_, time steps in the input sequence *w*_*back*_ = 24, time steps in the output sequence *w*_*ahead*_ = 24. We adopt a 24-hour time horizon because the day-ahead prediction of renewable generation is the most common requirement of smart grid management algorithms (*e.g*. Conte et al., 2020; Zhang et al., 2023; Conte et al., 2018; Michael et al., 2023), further considering the daily cyclical nature of PV generation.

Our proposed MATNet architecture and the entire training process are based on the PyTorch framework (Paszke et al., 2019). We trained all the models on a workstation with 1 NVIDIA A100 GPU for 200 epochs. The model weights and biases are updated using the Mean Squared Error (MSE) as a loss function, which measures the average squared difference between the predicted and the actual values. We used the Adam optimizer (Kingma and Ba, 2014) with a learning rate of 10^−3^ and with β_1_ and β_2_ equal to 0.9 and 0.999, respectively. We reduced the learning rate throughout training by a factor of 0.2 once learning stagnates, and no improvement is seen for a patience number of epochs equal to 20. The best-performing model on the validation set was used for final testing on the independent test set. The code is publicly available on GitHub.^1^

### 5.2 Evaluation metrics

To assess the quality of the proposed forecast model, we employed standard evaluation metrics commonly used in forecasting (Hyndman and Koehler, 2006), providing different insights on the prediction errors. In the following equations, *n* is the number of observations, *y*_*i*_ is the actual value of the *i*-th observation, and ŷ_*i*_ is the predicted value of the *i*-th observation:

- **Root mean squared error**: it is calculated as the square root of the mean of the squared differences between the predicted and actual values:
$$\begin{array}{l} \text{RMSE}=\sqrt{\frac{1}{n}\overset{n}{\underset{t=1}{\sum }}{\left(y_{t}-{ŷ}_{t}\right)}^{2}} \end{array}$$ (3)
- **Mean absolute error**: it measures the average absolute difference between the predicted and actual values:
$$\begin{array}{l} \text{MAE}=\frac{1}{n}\overset{n}{\underset{t=1}{\sum }}|y_{t}-{ŷ}_{t}| \end{array}$$ (4)
- **Weighted mean absolute percentage error**: it measures the accuracy of a forecast, where the importance of the forecast errors is weighted according to the size of the actual values being forecast:
$$\begin{array}{l} \text{wMAPE}=\frac{\sum_{t=1}^{n}|y_{t}-{ŷ}_{t}|}{\sum_{t=1}^{n}|y_{t}|} \end{array}$$ (5)
- **Mean absolute scaled error**: it measures the accuracy of a forecast, where the forecast errors are scaled by the mean absolute error of a naïve forecast:
$$\begin{array}{l} \text{MASE}=\frac{\frac{1}{n}\sum_{t=1}^{n}|y_{t}-{ŷ}_{t}|}{\frac{1}{n-1}\sum_{t=2}^{n}|y_{t}-y_{t-1}|} \end{array}$$ (6)

Each metric is in ${\mathbb{R}}_{0}^{+}$, where lower values indicate more accurate forecasts.

### 5.3 Competitors

We compare the performance of MATNet against four groups of competitors.

The first pool of competitors includes four well-established statistical time series forecasting models: AutoARIMA (Hyndman and Khandakar, 2008) and AutoCES (Svetunkov et al., 2022), both of which are statistical models that automatically select model parameters for each time series based on the Akaike information criterion (Sakamoto et al., 1986); NPTS (Fan and Yao, 2008), a non-parametric method based on local forecasting that assumes a non-parametric sampling distribution; and Theta (Assimakopoulos and Nikolopoulos, 2000), a dynamic forecasting technique that is particularly effective for non-seasonal or deseasonalized time series.

Second, we included the four approaches (Fentis et al., 2020; Kaur et al., 2021, 2023, 2022a) specific for the Ausgrid dataset, already introduced in section 2.

The third group of competitors includes several variations of our MATNet approach, where we substitute the self-attention mechanism with four alternative recurrent-based backbones, which share the same multi-level fusion and final fully connected layers, whilst the input embedding, positional encoding, and dense interpolation layers are no longer necessary. The purpose of this comparison is to assess the impact of self-attention on the model's performance relative to recurrent architectures. The four backbones we consider are: LSTM (Hochreiter and Schmidhuber, 1997), bi-directional LSTM (BiLSTM) (Graves and Schmidhuber, 2005), GRU (Chung et al., 2014), and BiGRU (Dong et al., 2016).

The final group of competitors includes two additional MATNet variants designed to evaluate the role of the DIL module. The first, *cDIL-based MATNet*, replaces the DIL with its non-trainable counterpart originally proposed in (Trask et al., 2015), as discussed in subsection 4.4. The second, *MATNet w/o DIL*, removes the interpolation layer and relies on last-token aggregation.

Note that we implemented all the methods except for the statistical models, which are provided by AutoGluon (Shchur et al., 2023), a Python AutoML framework for probabilistic time series forecasting. While AutoGluon supports probabilistic forecasts, we adapted these models for point forecasting by averaging their outputs. An overview of the main configuration settings adopted for all comparative models is provided in Appendix E.

### 5.4 Results and discussion

In this section, we comprehensively evaluate the performance of MATNet across multiple benchmark scenarios and analysis dimensions. We begin by reporting results on the Ausgrid benchmark and proceed to analyze the impact of different input modalities through ablation studies. We then assess the model's sensitivity to missing data, followed by an evaluation of cross-site zero-shot generalization on five external PV datasets. Finally, we examine the computational complexity of MATNet.

#### 5.4.1 Comparative analysis

Table 2 reports the performance in terms of RMSE, MAE, wMAPE, and MASE of our proposed model on the Ausgrid benchmark, followed by other thirteen rows presenting competitors' results, divided as described in the previous section.

**Table 2:** Comparative analysis of the proposed method for PV power production forecasting.

	Architecture	RMSE ↓	MAE ↓	wMAPE ↓	MASE ↓
	MATNet	**0.0445**	**0.0243**	**0.1672**	**0.4072**
Statistical based	NPTS	0.1450	0.0859	0.6483	1.6155
	AutoARIMA	0.1698	0.1017	0.6468	1.7075
	AutoCES	0.2014	0.1582	0.9847	2.6068
	Theta	0.1887	0.1530	1.0654	2.7107
Ausgrid benchmark	LsSVR (Fentis et al., 2020)	0.1469	0.0866	0.7526	1.7864
	Bayes BiLSTM(Kaur et al., 2021)	0.1262	0.0840	0.6867	1.6577
	α-β Bayes BiLSTM(Kaur et al., 2022a)	0.1319	0.0856	0.7573	1.7892
	VAE Bayes BiLSTM(Kaur et al., 2023)	0.1317	0.0860	0.7737	1.8189
MATNet variants	LSTM-based MATNet	0.0517	0.0288	0.1993	0.4842
	GRU-based MATNet	0.0505	0.0284	0.1976	0.4824
	BiLSTM-based MATNet	0.0510	0.0285	0.1949	0.4730
	BiGRU-based MATNet	0.0484	0.0271	0.1864	0.4538
	cDIL-based MATNet	0.1333	0.0812	0.7196	1.6933
	MATNet w/o DIL	0.0459	0.0255	0.1746	0.4249

In bold we highlight the best results for each metric.

Let us first examine MATNet's performance (first row in Table 2) against the four statistical time series forecasting methods (rows 2-5). The results indicate that MATNet outperforms these statistical models across all metrics, and all observed differences are statistically significant with a *p*-value < 0.001, according to the Diebold-Mariano test (Diebold and Mariano, 2002), which is always used hereinafter. These results are likely attributed to MATNet's ability to capture complex nonlinear relationships within the data, which traditional statistical methods struggle to model effectively. Statistical methods rely heavily on predefined assumptions about the data and fail to adapt to intricate temporal dependencies arising in PV power forecasting, particularly under varying weather conditions.

Next, we compare MATNet against the four approaches (rows 6–9) representing the current state-of-the-art on the same benchmark (Fentis et al., 2020; Kaur et al., 2021, 2023, 2022a)^2^: MATNet significantly outperforms these four state-of-the-art methods on all the evaluation metrics, with statistically significant differences at a *p*-value < 0.001. Since such four approaches are unimodal, we attribute MATNet's enhanced performance to its multimodal fusion that exploits interdependencies not captured by previous methods.

Turning our attention to the comparison with the four recurrent-based backbones (rows 10-13), we notice that our attention-based architecture outperforms all of them, and the differences are all statistically significant with a *p*-value < 0.01. Here the key advantage of MATNet lies in its self-attention mechanism, which dynamically focuses on the most relevant features, rather than relying solely on sequential dependencies. This flexibility allows MATNet to capture short-term and long-term patterns in PV power production, which is crucial for accurate forecasting under fluctuating conditions. Furthermore, it is worth noting that all four recurrent-based MATNet variants also statistically outperform the competitors included in the statistical and state-of-the-art approaches (*p*-values < 0.001). This fact highlights the effectiveness of the multi-level soft-attention multimodal strategy, which introduces substantial improvements in forecasting accuracy, regardless of the specific backbone used. Finally, we evaluate the performance of the last two MATNet variants (rows 14-15), both designed to assess the role of the dense interpolation layer. MATNet significantly outperforms these variants, with differences statistically significant at *p*-value < 0.001 and *p*-value < 0.01, respectively, highlighting the contribution of the DIL to forecasting accuracy. The cDIL-based variant performs comparably to the statistical and state-of-the-art baselines, likely due to the limited adaptability introduced by its fixed-weight interpolation. In contrast, the MATNet w/o DIL variant achieves performance levels similar to the recurrent-based alternatives, suggesting that although its removal reduces accuracy, the architecture still benefits from multimodal design and attention-based fusion.

**Figure 5:**
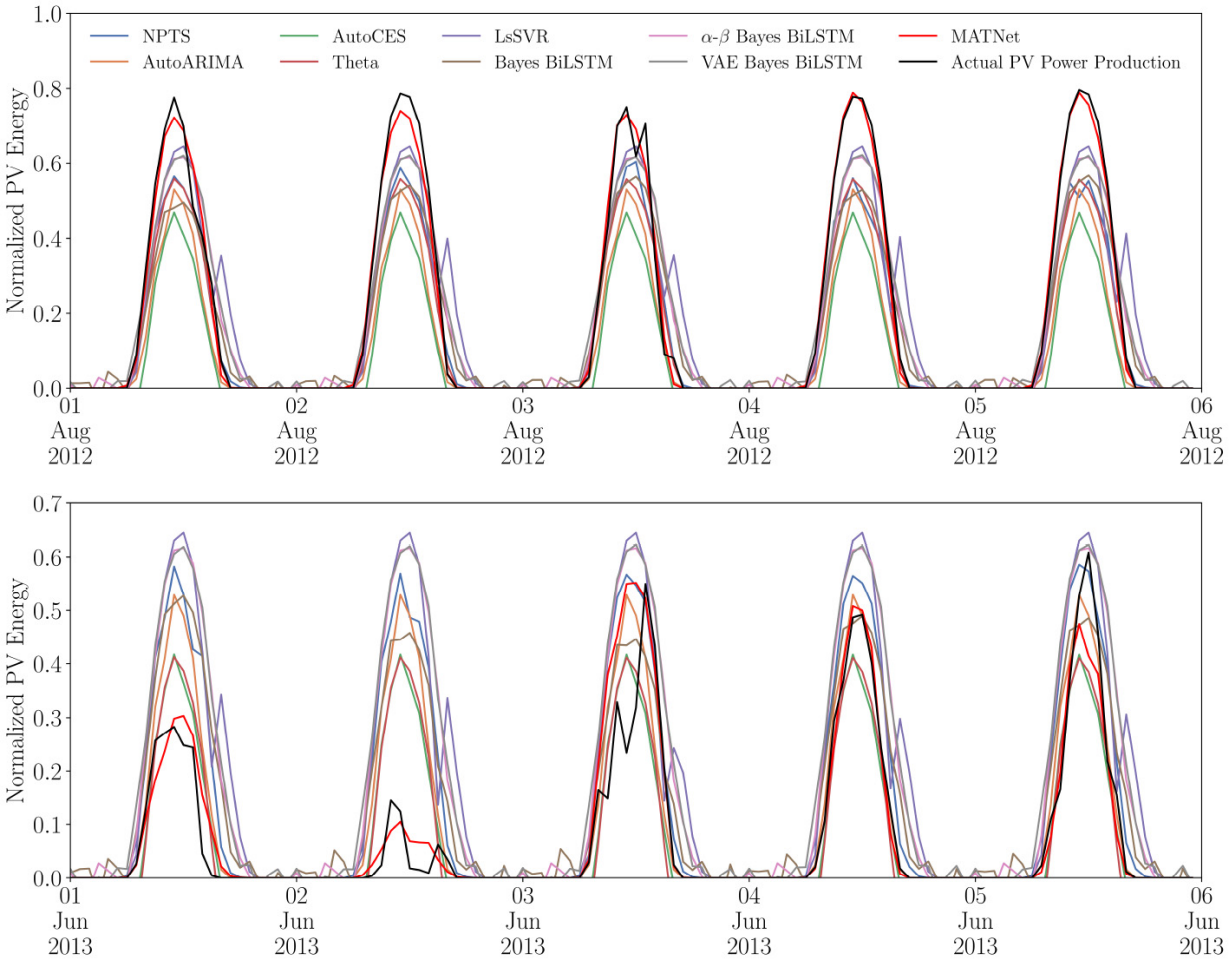
Comparison of hourly normalized PV power production (solid black line) with predictions from various models over two five-day periods (August 1-5, 2012 and June 1-5, 2013). The normalized PV energy is plotted on the y-axis, while the x-axis represents the date.

Figure 5 illustrates a comparison of hourly normalized PV power production against model predictions over two distinct five-day periods, specifically August 01-05, 2012, and June 01-05, 2013. The former, characterized by predominantly clear skies with occasional clouds and scattered rain, represents a relatively stable weather scenario. In contrast, the latter period, dominated by overcast skies and frequent rainfall, provides a more challenging test bed, enabling a comprehensive evaluation of model robustness under adverse weather conditions. For the sake of visualization, we show only the statistics-based and state-of-the-art approaches, excluding MATNet variants with recurrent-based backbones, as previous paragraphs have already established the superiority of MATNet's architecture over its other variants. In the first period reported in the top panel of Figure 5, MATNet demonstrates superior performance in consistently tracking the actual PV power production compared to other competitors. When examining the second period (bottom panel of Figure 5), MATNet still exhibits a smaller deviation from the actual values relative to other models. This is especially pronounced during rapid fluctuations in power production caused by dynamic weather patterns. MATNet's responsiveness and accuracy during these abrupt changes highlight its robustness, underscoring the efficacy of the attention mechanisms and multi-level joint fusion integrated into the forecasting process. Despite these advantages, it is worth noting that the predictions are not flawless, particularly on June 3 and 5, 2013, where MATNet exhibits some difficulty in closely following the actual power trends. This may be due to two key factors: first, the discrepancy between point-based weather measurements and the aggregate nature of the PV power production predictions, given that we consider 26 PV units distributed across an area of 75 square kilometers. We hypothesize that predicting individual PV unit outputs using point-specific weather data could improve the model's performance. Second, the need for a larger dataset to enhance the model's generalization capabilities. A stratified analysis of the test-set errors, reported in Appendix F, quantifies these effects: forecasting error increases under cloudy and rainy regimes and with the intensity of intra-day cloud transitions, while individual-day deviations reflect the combination of these factors with the point-to-aggregate mismatch noted above.

**Figure 6:**
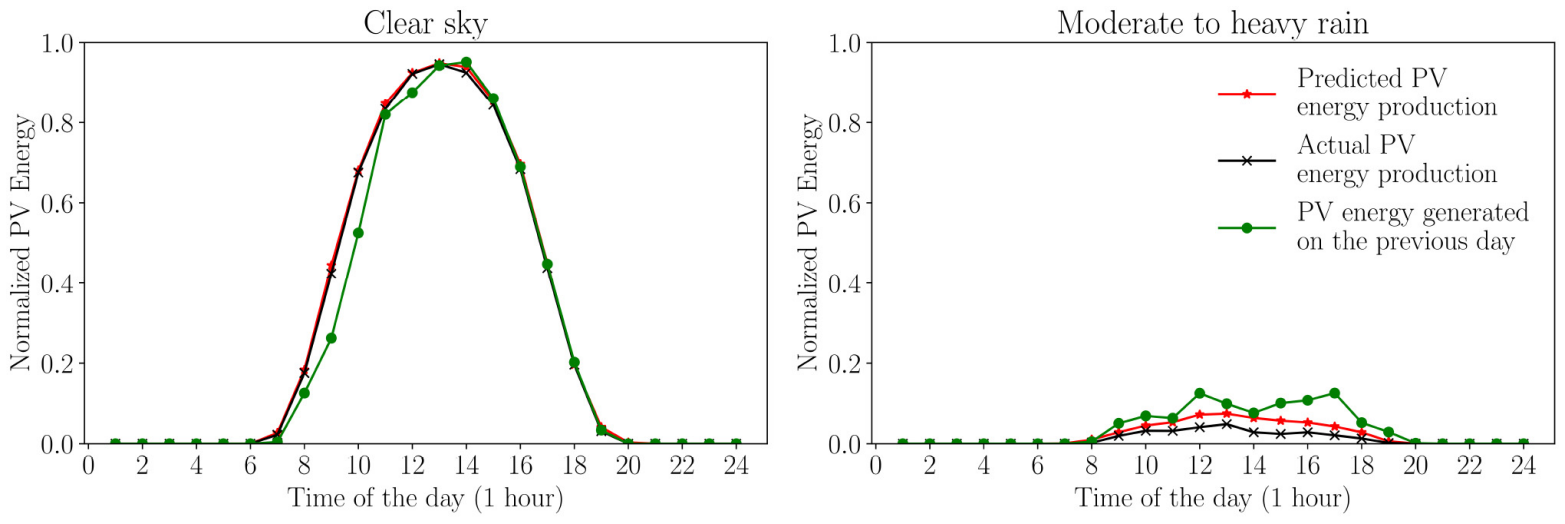
Forecasting results of MATNet on Ausgrid test set. On the **left** is the forecast for the best-performing day (2013-06-11), while on the **right** is the forecast for the worst-performing day (2013-01-28). Both predictions are evaluated using the MASE metric.

For the sake of completeness, Figure 6 shows the PV production forecasts of MATNet for the best-performing (left) and worst-performing (right) day according to the MASE metric. In black is the actual production curve and in red is the PV production forecast curve; besides, to provide additional context, we have also included the PV generation from the previous day, represented by the green curve. The plot on the left reveals the effectiveness of our MATNet on its best-performing day, with a MASE score equal to 0.0613. Predominantly clear skies characterize this day, and the forecast almost perfectly follows the actual PV production. On the other hand, the graph on the right shows the worst-performing day with a MASE equal to 2.2028. This day corresponds to a weather regime dominated by *heavy-intensity rain*, observed in only 0.15% of the training timestamps and thus extremely rare in the training distribution. The rarity of such adverse-weather events limits the ability of the attention mechanism to learn robust cross-modal dependencies, resulting in systematic under- or over-scaling of the predicted magnitudes despite the preservation of the diurnal trend. Extending the training period to additional years would broaden weather coverage and increase exposure to rare patterns, mitigating this limitation. Even under these challenging conditions, the forecast qualitatively reproduces the temporal evolution of PV production, highlighting the resilience of MATNet, which continues to outperform state-of-the-art methods that typically experience a decline in performance during adverse weather situations. The difference between the red (forecast) and green (previous day's production) patterns also highlights that the forecast is not solely based on the previous day's production, demonstrating the positive impact of incorporating weather forecasts in our model.

##### 5.4.1.1 Analysis of multi-level fusion attention

**Figure 7:**
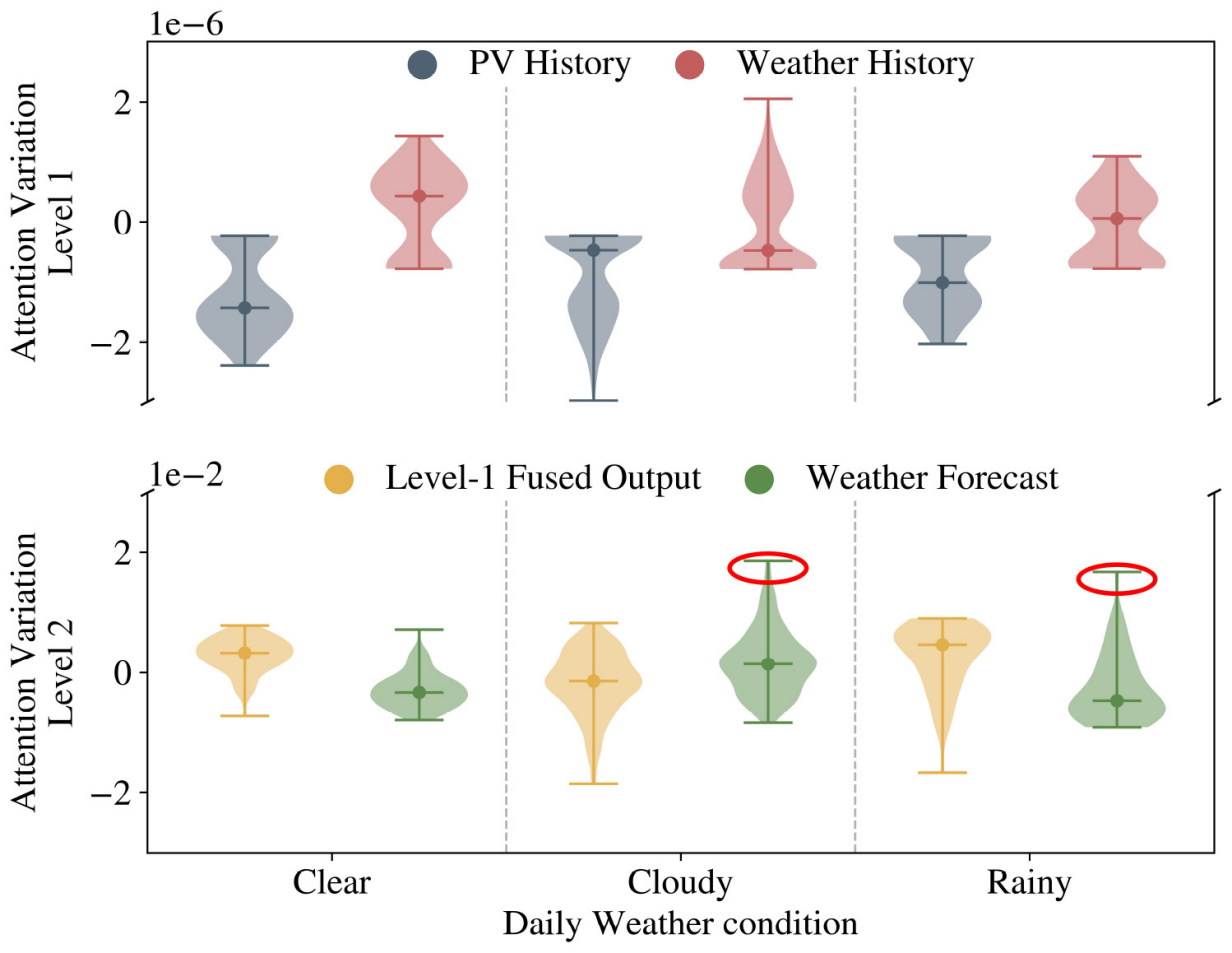
Violin plots of attention weight variations in the two-stage soft-attention fusion. **Top**: first-level fusion between historical photovoltaic (PV) and meteorological (Weather) inputs. **Bottom**: second-level fusion where the Level-1 fused output is further combined with the Weather Forecast. Each pair of violins is grouped by daily weather condition (*Clear, Cloudy*, and *Rainy*).

To further elucidate the interpretability of the proposed multi-level fusion mechanism and to reinforce the analysis of the worst-performing day, Figure 7 illustrates the distributions of attention weight variations learned by MATNet across the two fusion stages. The violin plots are grouped by daily weather condition (Clear, Cloudy, and Rainy), showing how the modality-specific attention weights evolve under different meteorological regimes.

In the first fusion level (top panel), which combines PV History and Weather History modalities, the variations are on the order of 10^−6^, indicating extremely stable attention behavior across samples. In absolute terms, the attention weights averaged across samples are consistently dominated by PV History (≈0.99), with Weather History contributing only marginally (≈0.01). Such quasi-stationary behavior demonstrates that MATNet primarily relies on historical PV information to infer short-term generation trends, regardless of the prevailing meteorological conditions. Since both first-stage inputs come from the same look-back window, in which PV production already reflects the concurrent meteorological conditions, the weather history is largely redundant, which accounts for the dominant weight of the PV branch. In contrast, in the second fusion level (bottom panel), where the *Level-1 fused output* is integrated with the Weather Forecast branch, the variations increase to the order of 10^−2^, revealing a stronger adaptive behavior. The future weather is the only input that informs the look-ahead window, so its weight rises when persistence from past production no longer extrapolates well. Under cloudy and rainy regimes, the distributions become broader and more asymmetric, as the model dynamically reweights modality contributions in response to forecast uncertainty. In particular, the regions of higher dispersion correspond to increased attention toward the Weather Forecast modality (see red circles in Figure 7), confirming that MATNet relies more heavily on predictive meteorological cues when PV dynamics become less deterministic.

These findings confirm the role of MATNet's multi-level soft-attention fusion: the first stage ensures stability by consistently leveraging historical PV information, whereas the second stage introduces adaptivity and cross-modal flexibility, enabling the model to maintain robustness and accuracy across diverse meteorological regimes. This conditional reliance on the forecast branch is consistent with the regime-dependent error reported in Appendix F, where accuracy decreases under cloudy and rainy conditions.

#### 5.4.2 Ablation analysis

To deepen the results, and given that MATNet is a multimodal architecture incorporating heterogeneous input sources (i.e., PV production data, weather history, and weather forecasts), we performed six ablation studies by systematically excluding one or two of these input modalities. In other words, for each study, we turned off the input branch corresponding to the removed data source, allowing us to evaluate how the model would perform without it. The results of this analysis provide valuable insights into each input modality's importance, helping us to assess the effectiveness of MATNet and its robustness in scenarios with missing modalities. The results are summarized in Table 3, where the first three columns indicate the input modalities used in each experiment. Straightforwardly, the last row corresponds to the full MATNet configuration with all modalities enabled.

**Table 3:** Ablation study results from the Ausgrid test set comparing the MATNet model's performance with various input branches disabled.

pv Production	Weather History	Weather Forecast	RMSE ↓	MAE ↓	wMAPE ↓	MASE ↓
✓	✗	✗	0.1078	0.0629	0.5689	1.3433
✗	✓	✗	0.1381	0.0863	0.7508	1.7756
✗	✗	✓	0.0815	0.0547	0.3986	0.9834
✓	✓	✗	0.1024	0.0592	0.4972	1.1916
✓	✗	✓	0.0455	0.0247	0.1714	0.4159
✗	✓	✓	0.0454	0.0249	0.1678	0.4091
✓	✓	✓	**0.0445**	**0.0243**	**0.1672**	**0.4072**

In bold we highlight the best results per metric.

Focusing on the unimodal configurations (rows 1-3), we observe that PV production data and weather forecasts outperform all the statistical and state-of-the-art competitors across all metrics, with statistically significant differences (p-value < 0.01). The weather history modality also outperforms statistical models but performs comparably to the state-of-the-art methods. This outcome may be attributed to the nature of the historical weather data, which, while informative, lacks the predictive power of weather forecasts or PV data and may fail to capture rapid changes or anomalies effectively. Among the unimodal configurations, the best performance is achieved when using only the weather forecast data. This result underscores the pivotal role of forecasted weather conditions in determining the model's success.

Shifting our attention to the bimodal configurations (rows 4-6), we observe that all bimodal combinations outperform the competitors across all metrics. These results further reinforce the notion that incorporating multiple heterogeneous data sources substantially enhances the model's performance. Notably, configurations that include the weather forecast modality (rows 5 and 6) consistently show significant improvements over all the statistical and state-of-the-art competitors, with *p*-values < 0.001, and their performance is comparable to the full MATNet model, with no statistically significant differences (*p*-values >0.05).

These findings suggest that MATNet maintains strong performance, even when one or more input modalities are unavailable, except for the unimodal configuration involving only the weather history modality. This robustness ensures that, in practical applications where data may be incomplete or unavailable, MATNet continues to function effectively and reliably in most scenarios.

### 5.4.3 Sensitivity analysis on missing data

**Figure 8:**
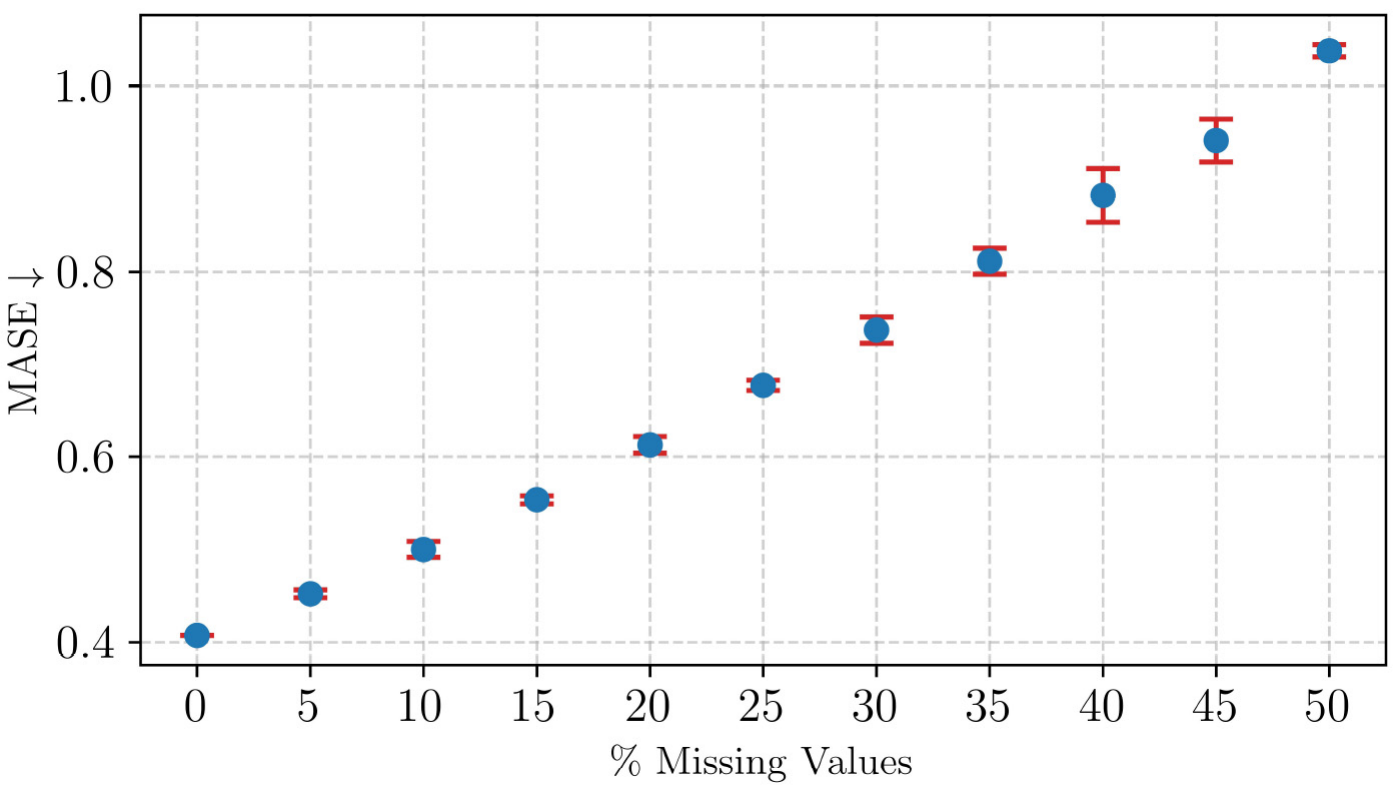
Evaluation of MATNet's robustness to missing input data. Each point reports the MASE on the Ausgrid test set when a given percentage of input features is randomly masked (from 5% to 50%, in steps of 5%) across all input modalities.

We evaluated MATNet's robustness under conditions of incomplete input data, a common challenge in real-world deployments due to sensor faults, transmission errors, or data acquisition delays. To simulate such scenarios, we applied value masking during inference by randomly setting a growing proportion of input features to zero, ranging from 5% to 50% in increments of 5%. Figure 8 reports the MASE on the Ausgrid test set as a function of the proportion of masked inputs. MATNet maintains relatively stable performance up to approximately 20% missing data, with only a modest increase in error. Beyond this threshold, performance degrades progressively. Around 35% missingness leads to a doubling of MASE relative to the fully observed baseline, yet without abrupt failure, highlighting the model's ability to degrade gracefully under increasing input corruption. It is worth noting that even with 50% of the input features missing, MATNet still outperforms all external baseline models operating under complete data availability (Table 2), including both statistical methods and Ausgrid-specific deep learning approaches. We attribute MATNet's resilience to its multimodal design: unlike competing models, which are univariate and limited in their ability to capture complex environmental dependencies, MATNet integrates multiple data sources, enabling it to infer missing information through cross-modal relationships.

### 5.4.4 Cross-site zero-shot generalization

To further assess the generalizability and robustness of MATNet, we conducted a cross-site zero-shot generalization experiment, testing the model on five publicly available PV generation datasets, whose characteristics are summarized in Table 4. These datasets differ from Ausgrid in multiple aspects. First, they vary significantly in terms of installed PV capacity, ranging from large-scale facilities (e.g., Hebei with 6600 kWp) to small residential setups (e.g., Austin with 6.3 kWp), whereas Ausgrid represents an aggregated signal from 26 households with moderate capacity. Second, the datasets are geographically diverse, covering four continents and spanning different hemispheres, which results in distinct seasonal patterns. Third, the underlying climate conditions are highly heterogeneous, ranging from semi-arid (BSk) to humid subtropical (Cfa), in contrast to the Cfa climate zone of the Ausgrid dataset. These substantial differences ensure that each external dataset represents a distinct operational regime, allowing us to assess the zero-shot generalization ability of MATNet under varying environmental, technical, and climatic conditions.

**Table 4:** External validation sites with location, period, Köppen-Geiger climate type (BSk, cold semi-arid; Csa, Mediterranean hot summer; Cwa, humid subtropical with dry winter; Csb, Mediterranean warm summer; Cfa, humid subtropical with no dry season), and PV capacity (kWp).

Dataset	Location	Latitude	Longitude	From date	To date	Köppen-Geiger	Installed Power (kWp)
Yao et al. (2021)	Hebei	38.047	114.951	08/16/18	06/13/19	BSk	6600
Sarmas et al. (2025)	Lisbon	38.728	-9.138	01/01/19	12/31/19	Csa	46
Lin et al. (2025)	Hong Kong	22.340	114.260	08/24/21	08/24/22	Cwa	16.22
Nie et al. (2023)	Stanford	37.427	-122.174	01/01/18	12/31/18	Csb	30
Pecan Street Inc. (2009)	Austin	30.267	-97.743	01/01/18	12/31/18	Cfa	6.3

Table 5 reports the results of the external validation in terms of RMSE and MAE for MATNet and its backbone variants across the five sites. The best-performing value for each metric and dataset is highlighted in bold, while the AvgWins row indicates the percentage of cases in which each method achieves the best result. MATNet exhibits strong zero-shot generalization, consistently achieving the lowest errors on the majority of external sites, attaining the best performance in 65% of cases. The model maintains high predictive accuracy despite substantial differences in site characteristics, climate patterns, and PV capacity. In comparison, recurrent-based variants, such as the LSTM-based MATNet, achieve competitive results on a single dataset (e.g., 15% of best scores) but exhibit inferior and less consistent performance across datasets. The MATNet w/o DIL variant wins in 20% of cases, confirming that removing the interpolation layer leads to a performance drop, thus validating the importance of temporal densification. Notably, the cDIL-based MATNet variant exhibits the highest error rates across all sites, underscoring the critical role of the trainable dense interpolation layer for effective temporal modeling in diverse scenarios.

**Table 5:** Performance comparison of MATNet and its backbone variants across five external benchmark datasets.

Methods	MATNet		LSTM-based MATNet		GRU-based MATNet		BiGRU-based MATNet		BiLSTM-based MATNet		cDIL-based MATNet		MATNet w/o DIL	
Metric	RMSE ↓	MAE ↓	RMSE ↓	MAE ↓	RMSE ↓	MAE ↓	RMSE ↓	MAE ↓	RMSE ↓	MAE ↓	RMSE ↓	MAE ↓	RMSE ↓	MAE ↓
Hebei	**0.0763**	**0.0438**	0.0853	0.0495	0.0857	0.0486	0.0861	0.0492	0.0787	0.0454	0.1212	0.0763	0.0785	0.0445
Lisbon	**0.1768**	**0.1099**	0.1847	0.1153	0.2146	0.1352	0.1942	0.1216	0.1835	0.1154	0.2284	0.1446	0.1848	0.1141
Hong Kong	0.1089	**0.0610**	**0.1071**	**0.0610**	0.1262	0.0726	0.1140	0.0654	0.1173	0.0675	0.1786	0.1076	0.1088	0.0611
Stanford	0.0668	0.0398	0.0710	0.0426	0.0688	0.0413	0.0680	0.0403	0.0693	0.0412	0.1229	0.0792	**0.0613**	**0.0360**
Austin	**0.0832**	**0.0481**	0.0912	0.0529	0.0998	0.0588	0.0935	0.0550	0.0923	0.0542	0.1593	0.0961	0.0847	0.0487
AvgWins	65%		15%		0%		0%		0%		0%		20%	

Results are reported as RMSE and MAE (lower is better). Bold values indicate the best performance per metric and dataset; AvgWins denotes the percentage of best results for each method.

These results underscore the robustness and transferability of MATNet, demonstrating its capacity to generalize effectively to previously unseen and heterogeneous PV generation environments without requiring site-specific adaptation. The cross-site evaluation further highlights MATNet's potential for practical deployment in real-world energy management scenarios across diverse operational contexts.

### 5.4.5 Computational complexity analysis

**Figure 9:**
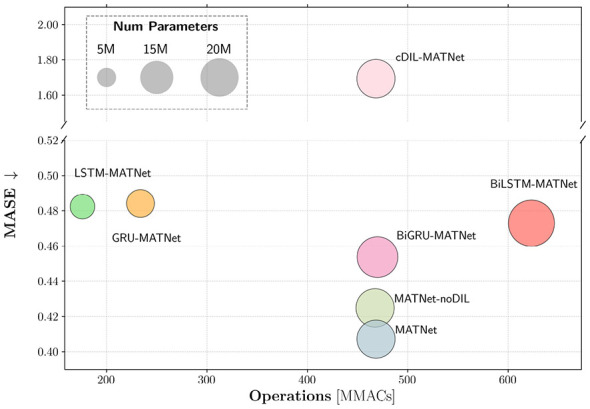
Comparison of MATNet and its backbone variants in terms of MASE (y-axis) and computational cost in MMACs (x-axis). Bubble size reflects the number of parameters, as shown by the reference bubbles in the top left (5M, 15M, and 20M parameters).

In practical forecasting applications, computational complexity is a key consideration alongside predictive accuracy. Figure 9 illustrates the trade-off between computational cost and forecasting accuracy for MATNet and its backbone variants, summarizing the number of trainable parameters, inference-time MMACs, and test accuracy in terms of MASE.

MATNet achieves the lowest MASE with a moderate computational cost, making it the most efficient architecture among those considered. Recurrent-based models, such as BiLSTM-MATNet and BiGRU-MATNet, require significantly more operations and parameters while delivering inferior accuracy. The cDIL-based MATNet, in contrast, exhibits both higher computational cost and the highest MASE among all variants. Traditional recurrent architectures like LSTM-MATNet and GRU-MATNet offer lower computational demands but do not achieve the same accuracy as MATNet. This indicates that, despite their efficiency, they are less suitable for high-fidelity PV forecasting in diverse scenarios. MATNet w/o DIL achieves a slightly lower computational cost than the full MATNet. However, this minor gain in efficiency comes with a drop in accuracy, underscoring the contribution of the DIL module to MATNet's superior forecasting performance. These results confirm that MATNet provides the best balance between accuracy and computational efficiency, supporting its deployment in real-world energy forecasting applications where both performance and resource constraints must be considered.

## 6 Conclusion

In this work, we proposed MATNet, a novel multimodal self-attention architecture for multi-step, day-ahead PV power generation forecasting. The architecture leverages historical PV production data, historical weather data, and weather forecasts as inputs, and integrates these heterogeneous modalities through a multi-level soft-attention fusion strategy that effectively combines information at different levels of abstraction.

We extensively evaluated MATNet on the Ausgrid benchmark dataset, where it significantly outperformed thirteen baseline models, including statistical, machine learning, and deep learning approaches. Specifically, MATNet achieved a RMSE of 0.0445 and a MASE of 0.4072, corresponding to relative improvements of approximately 65% and 75%, respectively, compared to the best-performing external baseline (Bayes BiLSTM). The model's ability to capture temporal and cross-modal dependencies through its soft-attention fusion framework was key to achieving this superior predictive performance. We further assessed MATNet's robustness to incomplete inputs through a sensitivity analysis on missing data. MATNet maintained stable performance up to 20% missingness and showed graceful degradation under higher corruption levels. Even under 50% missingness, the model outperformed all external baselines, confirming its ability to recover informative signals via cross-modal inference. Furthermore, MATNet demonstrated strong generalization capabilities through a cross-site zero-shot evaluation conducted on five external PV datasets with diverse operational contexts, climate types, and installed capacities. The model consistently achieved high forecasting accuracy under substantial domain shifts without requiring site-specific adaptation. In addition, our computational complexity analysis confirmed that MATNet provides an effective balance between predictive accuracy and resource efficiency, making it suitable for practical deployment in real-world forecasting applications where both performance and computational cost are critical considerations.

While the proposed method achieves remarkable forecasting accuracy, it relies on the integration of forecast and historical weather data to enhance PV power production predictions. It is worth recognizing that the acquisition of these data can involve costs in some cases. However, the services utilized in our research provide access to these data free of charge for household PV systems. Therefore, incorporating meteorological data does not impose any financial burden for the specific use case of household applications. We also note that, due to the lack of available historical forecast data for the study period, our model relies on a simulated forecast input derived from past weather observations. While this strategy introduces controlled perturbations in the future-weather inputs, it cannot reproduce the error structure of archived operational forecasts. In a practical deployment, this gap is removed by connecting the future-weather branch to an operational NWP provider, which the architecture already supports since the forecast is treated as a separate input stream. Moreover, the dataset used in this study was limited to 26 residential PV units with complete and high-quality data. While this selection ensured consistent and reliable measurements, it may also introduce a mild selection bias toward more stable systems, potentially reducing the model's generalizability to larger or noisier datasets. The missing-data sensitivity analysis in subsubsection 5.4.3 already shows that MATNet degrades gracefully under increasing input corruption, which suggests robustness to incomplete measurements. Future work will build on this by incorporating data imputation strategies and by extending the training to larger, more diverse PV datasets that include noisier and less curated systems, to further test the scalability and robustness of the proposed approach. A further consideration concerns long-term deployment. The mapping from meteorological covariates to PV generation is stable over a multi-year horizon, as confirmed by the cross-site evaluation in subsubsection 5.4.4, yet factors such as panel degradation, plant reconfiguration, or changes in the meteorological data provider can induce input drift. Monitoring this drift and retraining the model periodically would preserve forecasting accuracy over extended deployment.

To further improve the proposed method by making it more resilient, robust, and reliable, future work is directed toward developing an adaptive joint fusion layer. The idea is to dynamically weight the contribution of the various input modalities to provide more weight to the weather forecast branch during adverse conditions. In this way, the model can better adjust its predictions to follow the typical silhouette of PV generation in clear weather, while giving more weight to the branch of the weather forecast in case of adverse conditions. Besides, another worthy future direction is the incorporation of the eXplainable AI paradigm into the proposed framework. By making the proposed framework interpretable, we can facilitate the integration of AI-based RES into the power systems, enabling energy stakeholders to make informed, evidence-based decisions. In this context, investigating feature importance and selection strategies could enhance interpretability and reveal the contribution of individual weather variables to the model's predictions, offering additional insights for decision-makers.

## Data Availability

The original contributions presented in the study are included in the article/Supplementary material, further inquiries can be directed to the corresponding author.

## Footnotes

1 The code is available at the following GitHub repository https://github.com/arco-group/MATNet.

2 It is worth noting that we re-implemented these methods to ensure consistency with our validation; therefore, the results in our study may differ from those originally reported.
