# Supplementary material for: MATNet: multi-level fusion transformer-based model for day-ahead PV generation forecasting
Source: Front Artif Intell. 2026 Jul 14;9:1891798. doi: 10.3389/frai.2026.1891798 (PMC13407617; doi:10.3389/frai.2026.1891798)
Supplement: Supplementary file 1 [file Supplementary_file_1.pdf]

Supplementary Material

APPENDIX A SELECTED AUSGRID CUSTOMERS

In this work, we only focused on 26 out of the 300 customers: 33, 47, 73, 87, 88, 110, 124, 144, 151, 153, 157, 163, 175, 176, 188, 200, 201, 207, 222, 240, 256, 259, 263, 272, 281, 293, for a total of 8 postal codes in Newcastle region. These have no missing values or artifacts due to inactivity, failures, or interruptions.

APPENDIX B SENSITIVITY ANALYSIS ON INPUT GRANULARITY

To assess the impact of data resolution, we conducted a comparative analysis across multiple input granularities ranging from 30 minutes to 6 hours (Figure S1 (a)).

To generate coarser resolutions, the data were aggregated according to the nature of each variable. Photovoltaic production was resampled following the same rule defined in Equation 1. For meteorological data, extensive variables over time (e.g., precipitation) were aggregated by summation, intensive variables (e.g., temperature, humidity, pressure, dew point, wind speed, cloud cover, GHI/DNI/DHI) by arithmetic mean, and directional variables (e.g., wind direction) by circular mean, while categorical attributes (e.g., weather description) were aggregated by mode.

The results indicate that MATNet maintains stable performance across different granularities, with errors gradually decreasing at coarser resolutions due to the smoothing of short-term fluctuations. The 1-hour resolution emerges as the most appropriate choice, providing a favorable trade-off between forecasting

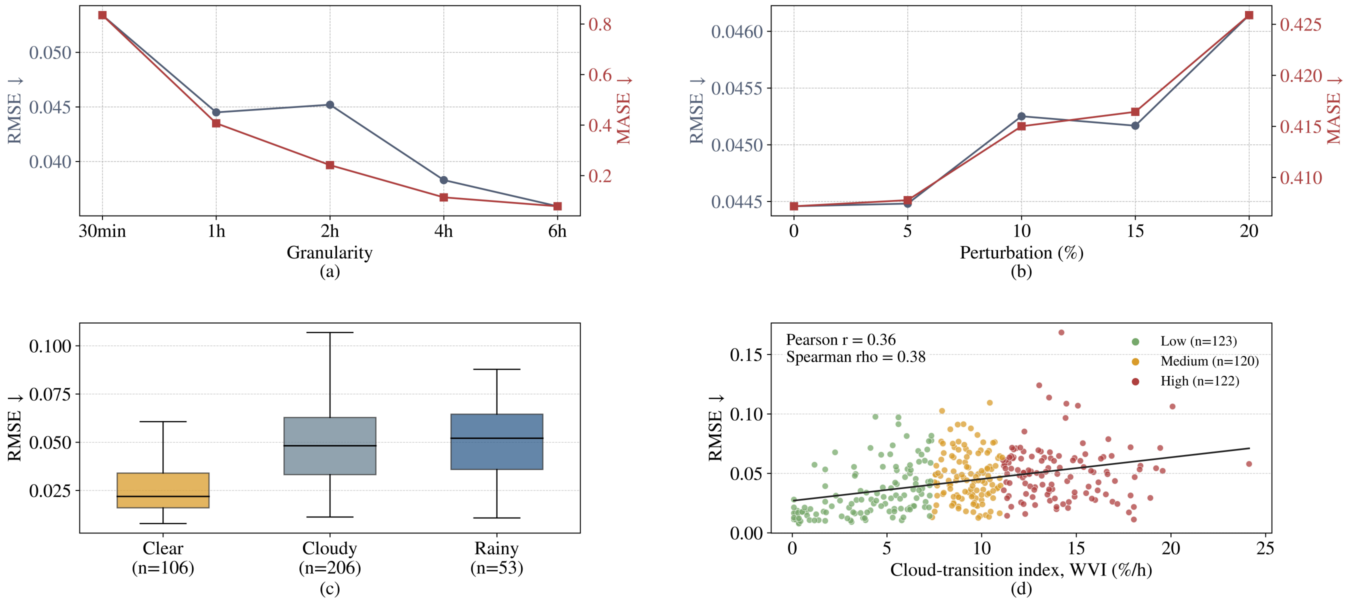

Figure S1. Sensitivity and stratified error analyses of MATNet. (a) Robustness across input time granularities (30 min to 6 h), with performance in RMSE (left axis) and MASE (right axis). (b) Noise sensitivity under increasing Gaussian perturbations (0–20%) applied to weather inputs, in RMSE (left axis) and MASE (right axis). (c) Per-day RMSE by daily weather regime (Clear, Cloudy, Rainy). (d) Per-day RMSE against the cloud-transition index WVI, with days grouped into Low, Medium, and High tertiles.

accuracy and temporal detail, while aligning with operational practices in energy markets and retaining sufficient information for day-ahead forecasting.

APPENDIX C WEATHER DESCRIPTION OF OPENWEATHERMAP

The following lists the various levels of the categorical *Weather Description* feature provided by the OpenWeatherMap API: *scattered clouds*, *few clouds*, *broken clouds*, *overcast clouds*, *sky is clear*, *light rain*, *thunderstorm*, *moderate rain*, *fog*, *light intensity shower rain*, *mist*, *haze*, *heavy intensity rain*, *light intensity drizzle*, *shower rain*, *smoke*, *thunderstorm with rain*, *proximity squalls*, *very heavy rain*, *light intensity drizzle rain*, *rain and drizzle*, *drizzle*.

APPENDIX D NOISE SENSITIVITY ANALYSIS

To evaluate the robustness of MATNet to uncertainty in weather forecasts, we conducted a sensitivity analysis by injecting Gaussian perturbations of increasing magnitude into the weather input data. Gaussian noise levels ranging from 0% to 20%, with 5% increments, were applied to meteorological features to simulate potential deviations between predicted and actual weather conditions (Figure S1 (b)).

The results indicate that MATNet maintains stable performance across all noise levels, exhibiting only a gradual degradation as perturbations increase. Specifically, model performance remains largely consistent up to 20% noise, with relative increases of 3.8% in RMSE and 4.6% in MASE compared to the noise-free condition. These findings confirm the robustness of MATNet against forecast uncertainty and support the realism of the 5% Gaussian noise adopted in the main experimental setup.

APPENDIX E BASELINE CONFIGURATIONS

Statistical baselines are implemented via AutoGluon (Shchur et al., 2023) v1.1.0 with default settings:

- **AutoARIMA**: `seasonal`=True, `stationary`=False.
- **AutoCES**: `model`=Z, `seasonal_period`=None.
- **NPTS**: `kernel_type`=Exponential, `use_seasonal_model`=True.
- **Theta**: `decomposition_type`=Multiplicative, `seasonal_period`=None.

The Ausgrid-specific baselines, introduced in Section 2, follow the original configurations (Fentis et al., 2020; Kaur et al., 2021, 2022, 2023):

- **LsSVR**: `kernel`=Radial Basis Function.
- **Bayes BiLSTM**: `optimizer`=Adam, `loss`=KL-divergence.
- α-β **Bayes BiLSTM**: $\alpha = 1.0$, $\beta = 2.0$.
- **VAE Bayes BiLSTM**: `latent_dim`=10, `loss`=ELBO.

All competitors are trained on the same splits and evaluated under identical forecasting settings as MATNet.

APPENDIX F STRATIFIED ERROR ANALYSIS

To characterize the conditions under which MATNet's accuracy decreases, we stratify the daily forecasting errors on the Ausgrid test set along two axes. The first is the daily weather regime, assigned to each test day from the dominant OpenWeatherMap weather description and grouped into Clear, Cloudy, and Rainy.

The second is the intra-day cloud variability, quantified through a cloud-transition index WVI, defined as the mean absolute hourly change in cloud cover:

$$\mathrm{WVI} = \frac{1}{T-1} \sum_{h=2}^{T} |c_h - c_{h-1}| \,, \tag{S1}$$

where c_h is the cloud cover at hour h and $T = 24$. We use cloud cover because it is the dominant driver of short-term PV variability. Days are split into Low, Medium, and High WVI tertiles.

Figure S1(c) and Figure S1(d) report the per-day RMSE for the two stratifications. Median RMSE increases from Clear (0.0219) to Cloudy (0.0482) and Rainy (0.0521), with $n = 106$, 206, and 53 days, respectively. The Cloudy and Rainy distributions overlap, indicating that the presence of clouds, rather than their precise type, drives most of the increase relative to clear days. Per-day RMSE also increases with WVI (Pearson $r = 0.36$, Spearman $\rho = 0.38$, $n = 365$), confirming that days with stronger intra-day cloud transitions are harder to forecast. The moderate correlation indicates that intra-day variability is one of several error sources, alongside the weather regime and the point-to-aggregate mismatch discussed in Section 5.4.1. Individual days can therefore deviate from both trends when other factors dominate.

REFERENCES

Fentis, A., Lytridis, C., Kaburlasos, V. G., Vrochidou, E., Pachidis, T., Bahatti, E., et al. (2020). A machine learning based approach for next-day photovoltaic power forecasting. In *2020 Fourth International Conference On Intelligent Computing in Data Sciences (ICDS)* (IEEE), 1–8

Kaur, D., Islam, S. N., Mahmud, M., et al. (2022). A bayesian deep learning technique for multi-step ahead solar generation forecasting. *arXiv preprint arXiv:2203.11379*

Kaur, D., Islam, S. N., and Mahmud, M. A. (2021). A bayesian probabilistic technique for multi-step ahead renewable generation forecasting. In *2021 IEEE 2nd International Conference on Smart Technologies for Power, Energy and Control (STPEC)* (IEEE), 1–6

Kaur, D., Islam, S. N., Mahmud, M. A., Haque, M. E., and Anwar, A. (2023). A vae-bayesian deep learning scheme for solar power generation forecasting based on dimensionality reduction. *Energy and AI* 14, 100279

Shchur, O., Turkmen, A. C., Erickson, N., Shen, H., Shirkov, A., Hu, T., et al. (2023). Autogluon–timeseries: Automl for probabilistic time series forecasting. In *International Conference on Automated Machine Learning* (PMLR), 9–1
